# A Complete Coverage Path Planning Algorithm for Lawn Mowing Robots Based on Deep Reinforcement Learning

**DOI:** 10.3390/s25020416

**Published:** 2025-01-12

**Authors:** Ying Chen, Zhe-Ming Lu, Jia-Lin Cui, Hao Luo, Yang-Ming Zheng

**Affiliations:** 1 Center for Generic Aerospace Technology, Huanjiang Laboratory, Zhuji 311816, China; nampl@zju.edu.cn (Y.C.); luohao@zju.edu.cn (H.L.); zymsun2002@zju.edu.cn (Y.-M.Z.); 2 School of Aeronautics and Astronautics, Zhejiang University, Hangzhou 310027, China; 3 School of Information Science and Engineering, NingboTech University, Ningbo 315100, China

**Keywords:** path planning, complete coverage path planning, reward function, curiosity-driven exploration, dynamic ε-greedy strategy, dynamic environments, training stability

## Abstract

This paper introduces Re-DQN, a deep reinforcement learning-based algorithm for comprehensive coverage path planning in lawn mowing robots. In the fields of smart homes and agricultural automation, lawn mowing robots are rapidly gaining popularity to reduce the demand for manual labor. The algorithm introduces a new exploration mechanism, combined with an intrinsic reward function based on state novelty and a dynamic input structure, effectively enhancing the robot’s adaptability and path optimization capabilities in dynamic environments. In particular, Re-DQN improves the stability of the training process through a dynamic incentive layer and achieves more comprehensive area coverage and shorter planning times in high-dimensional continuous state spaces. Simulation results show that Re-DQN outperforms the other algorithms in terms of performance, convergence speed, and stability, making it a robust solution for comprehensive coverage path planning. Future work will focus on testing and optimizing Re-DQN in more complex environments and exploring its application in multi-robot systems to enhance collaboration and communication.

## 1. Introduction

With the rapid advancement of science and technology, mobile robots have become integral to various applications, including infrastructure inspection, rescue operations, and environmental maintenance tasks such as lawn mowing. A critical aspect of these applications is the need for complete coverage path planning (CCPP), which ensures that the robot can navigate and cover every part of a designated area. This requirement is essential for tasks where thoroughness is paramount, such as cleaning, inspection, and agricultural operations. The complexity of environments and the need for optimization and adaptability to different surfaces and obstacles make this an ongoing research challenge.

Traditional complete coverage path planning methods can be broadly categorized into the following types: partition-based, graph theory-based, and artificial intelligence-based methods. Choset [1] proposed that the CCPP algorithm can be divided into two categories: “online” and “offline”. The “offline” approach assumes that environmental factors are known, including the shape and area of the coverage region as well as the distribution of obstacles. In contrast, the “online” approach uses sensors equipped on the device to perform real-time scanning of the target environment when environmental information is completely or partially unknown. In the context of CCPP, a dynamic environment can be defined as one that contains real-time changing obstacles, unpredictable terrain variations, or other environmental conditions that may affect the robot’s path selection and navigation. Such an environment requires algorithms to perceive changes in real-time and adjust the path accordingly to ensure effective coverage of the target area without collisions or redundant coverage. This work specifically focuses on the “online” approach, highlighting its unique ability to adapt in real-time to dynamic environments and ensure efficient coverage under changing conditions. The subsequent sections will delve deeper into the online strategy and its practical application.

Latombe’s trapezoidal decomposition [2] splits the non-obstacle area into trapezoids [3], simplifying path planning but causing redundancy and inefficiency for irregular shapes. Choset’s boustrophedon method [4] reduces subregions for shorter paths [5]. As the first two use only vertical/horizontal cuts, Huang proposed the variable cutting direction method [6] to minimize robot turns.

Then came the grid method, first proposed by Elfes and Moravec [7]. Its coverage principle is to find an optimal non-repetitive path through all free grid cells. Based on it, Hodgkin and Huxley proposed the H-H model [8]. Grossberg introduced a neural dynamic network model [9]. Gabriely et al. [10] presented the spiral grid coverage method (Spiral-STC). Gonzalez et al. improved Spiral-STC by including “partially occupied” cells in the outer spiral for full area coverage [11]. Choi et al., using historical sensor data, introduced a map coordinate assignment to reduce robot turns [12].

As neural networks emerged, the field of path planning received new opportunities. Luo et al. [13] and Yang and Luo [14] used a neural net for CCPP in floor cleaning. DFS [15] and Q-learning [16] are common. DFS ignores path length, leading to long paths and inefficiency. Q-learning needs many samples and long training for good results.

A* [17] and RRT [18] are classic path planning algorithms that are widely used. However, the A* algorithm is not suitable for dynamic scenarios, and the Rapidly Exploring Random Tree (RRT) algorithm cannot guarantee an optimal path because it is heuristic. Meanwhile, the time-consuming calculations involved in high-dimensional maps [19], along with their poor generalization capabilities, have emerged as the main drawbacks for these algorithms.

With the emergence of the Deep Q Network (DQN), which combines deep learning and reinforcement learning, robotic path planning has undergone a profound transformation [20,21,22]. In particular, advances such as Double DQN (DDQN) [23,24] have further enhanced the capability of DQN to learn effective strategies in complex environments. These advances enable agents to navigate and plan optimal routes more efficiently in dynamic environments, addressing the shortcomings of traditional algorithms.

In dynamic environments, many studies have explored how to effectively handle real-time changing obstacles. For example, ref. [25] presented a path planning method for dynamic obstacles, which includes a reward function with penalty terms for the training process and employs a strategy of randomly setting starting and target points to increase the diversity of the training environment. Additionally, ref. [26] discussed a grid-based method suitable for obstacle localization and path planning, introducing a “shortest distance first” strategy to reduce the path length for drones reaching their targets. Ref. [27] investigated a multi-step update strategy that brings the Q network closer to the target value, which is particularly important when dealing with dynamic obstacles.

Multi-agent reinforcement learning (MARL) is an important branch of reinforcement learning (RL) and shows significant advantages in complex system tasks such as complete coverage path planning (CCPP) and searching tasks.

Wang et al. [28] automatically designed the trigger conditions for action advising based on genetic programming (GP) to optimize the collaborative decision-making of multiple agents and improve the adaptability in dynamic environments. Yuan et al.’s two-stage planning method [29] transforms the coverage path planning problem into an optimal grid selection problem, providing ideas for optimizing the path planning of a single agent. Ramezani et al. [30] constructed a fault-tolerant framework to ensure the system performance when some agents fail, emphasizing the importance of algorithm robustness.

Although this paper focuses on the path planning of a single agent, the achievements of MARL methods provide references for single-agent research in terms of collaboration, environmental adaptation, and reliability, which is helpful for expanding the single-agent algorithm to multi-agent scenarios and optimizing the path planning algorithm.

This paper introduces a novel algorithm called Re-DQN, where ‘Re’ stands for ‘Reinforced’, indicating that this algorithm is an improved and enhanced deep reinforcement learning method based on traditional DQN, which is designed to overcome the limitations of conventional path planning methods. Our Re-DQN utilizes deep reinforcement learning to enhance the efficiency, stability, and adaptability of lawn mowing robots operating in dynamic environments.

The rest of this paper is organized as follows. Section 2 describes the modeling of the robot and its environment, including considerations for obstacle avoidance and map preprocessing. Section 3 discusses the DQN algorithm and introduces the enhancements made in the Re-DQN algorithm. Section 4 compares and discusses the performance of the Re-DQN algorithm against traditional DQN. Section 5 summarizes the findings and discusses potential directions for future research.

## 2. Workspace Modeling and Robotic System Overview

This section first elaborates on the model for coverage path planning, which encompasses the robot model and the environment model. Then, it presents the design of the robot system, encompassing both the hardware and software platform setup. Consequently, this provides a solid foundation for subsequent research on localization and coverage algorithms.

**Figure 1:**
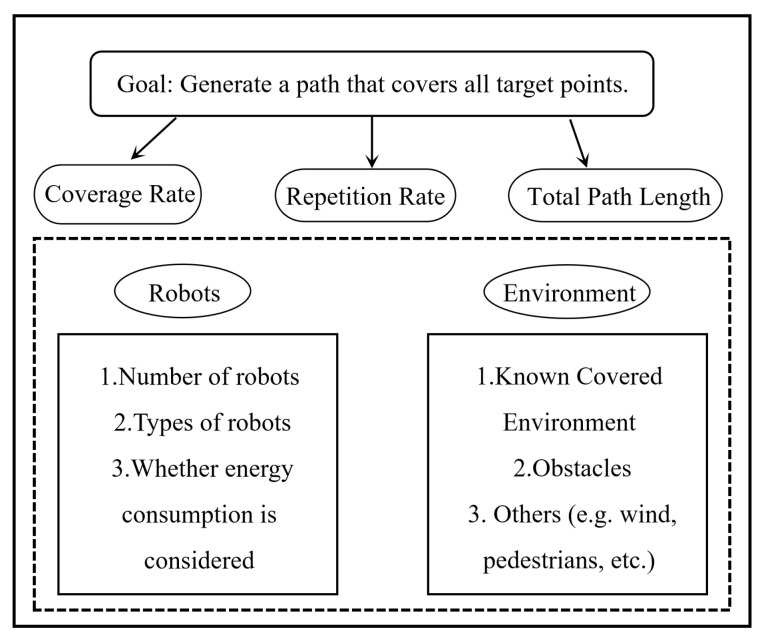
Overview of the CCPP problem.

An overview of the complete coverage path planning problem is shown in Figure 1. The objective is to generate a path that can cover all target points. The generated coverage path should be optimal to ensure a low repetition rate and high coverage efficiency.

### 2.1. Hardware and Software Architecture

**Figure 2:**
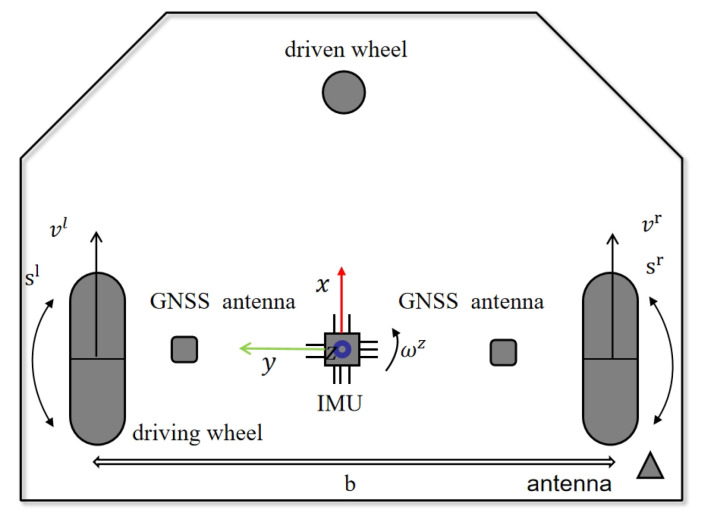
Key components and parameters of the robot.

Figure 2 shows the main movement components and sensor layout of the mower robot. First, the GNSS antennas on the left and right sides are used to receive GPS signals. These antennas, combined with RTK technology, provide high-precision positioning information for the mower robot.

The IMU, marked as a blue square in the diagram, is installed near the robot’s center of rotation to measure posture changes. The robot has a front swivel caster and two rear wheels driven by independent motors. With labeled linear and rotational speeds of the wheels, and a wheel track variable b affecting turning radius and maneuverability, along with its own coordinate system, this design enables autonomous navigation and precise control in complex outdoor environments.

#### 2.1.1. Integrated Hardware and Software System

The lawn mowing robot integrates hardware and software systems to ensure precise and autonomous operation. The hardware components include a power drive, MCU, IMU, two GNSS receivers, and UHF/VHF radio modules. The MCU is responsible for controlling the drive system and processing feedback from the wheel encoders, while the IMU and GNSS work together with RTK correction data received through the UHF/VHF module to further enhance positioning accuracy. The software architecture consists of Modbus, IMU and GNSS decoders, control modules, and positioning modules. These modules enable the real-time processing of sensor data, path planning, and accurate navigation, ensuring the robot can autonomously perform the mowing tasks along the planned route.

#### 2.1.2. Base Station Overview

The base station of the mowing robot system undertakes the responsibilities of path planning and data uploading. It acquires environmental position data via a GNSS receiver, calculates the robot’s path, and sends this information to the robot’s database for the robot to follow during task execution. Moreover, the base station provides RTK correction data through the UHF/VHF radio module to improve the robot’s positioning accuracy.

**Figure 3:**
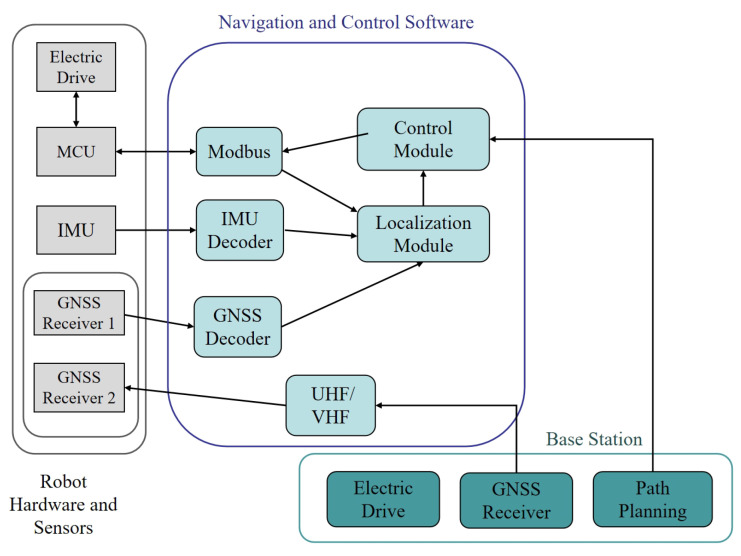
Overview of the system architecture.

As shown in Figure 3, the workflow encompasses path planning, real-time positioning, navigation and control, and data feedback and correction. The base station plans the path and transmits it to the robot. The robot uses sensors like IMU and GNSS to update its position. The control module adjusts motion commands based on error and controls the drive system via the MCU for accurate path following. Meanwhile, the robot receives RTK correction signals from the base station via UHF/VHF radio for high-precision positioning.

### 2.2. Simplified Robot Modeling

When the lawn mowing robot navigates within its designated workspace, it is imperative to prevent collisions with obstacles or the boundaries of the map. This requires a meticulous consideration of the robot’s dimensions and contours. In our work, we opt not to pursue real-time modeling for the grass-cutting robot due to concerns over high computational resource consumption and low path planning efficiency. Real-time modeling involves continuously updating the robot’s state and environmental information, which can demand significant computational resources, particularly in complex environments and large-scale work areas. Therefore, to avoid the aforementioned drawbacks, we opt to inflate obstacles to the size of the grass-cutting robot, ensuring their width equals the robot’s radius [31,32]. The obstacle cells in the map are enlarged by the robot’s radius $r_{r}$, which is defined as the distance from the robot’s center to its furthest perimeter point. For additional safety, the obstacle cells are actually enlarged by a radius $r_{obs}=r_{r}+d_{min}$, where $d_{min}$ is the minimum distance between the robot and the obstacle. By enlarging the obstacles to $r_{obs}$, the robot can be treated as a point-like vehicle.

**Figure 4:**
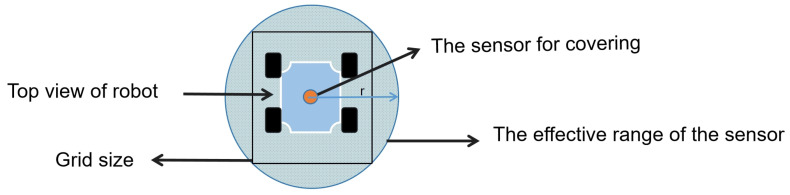
The relationship between the grid size and the sensor range.

This approach simplifies the irregular shape of the grass-cutting robot into a circular form as shown in Figure 4. Consequently, when calculating paths near the inflated obstacles, the algorithm ensures that the robot’s circular model does not intersect with any obstacles, thereby guaranteeing collision-free movement of the grass-cutting robot [33].

Designing the agent as a circle has multiple advantages: Firstly, collision detection is simplified. That is, when calculating collisions between the agent and obstacles, it is only necessary to compare the distance from the agent’s center to the obstacle’s boundary to see if it is less than or equal to the radius. This collision detection algorithm is more efficient compared to complex shapes. Secondly, coverage efficiency is high. That is, in CCPP, a circular agent can move and rotate more smoothly, effectively covering the area and reducing redundant coverage and omissions. Lastly, calculations are simplified. That is, to determine if a point is within the agent’s coverage area, it is only necessary to calculate the distance from the point to the agent’s center and compare it to the radius, making geometric calculations more straightforward.

### 2.3. Workspace Modeling and Preprocessing

In CCPP, the resolution of the grid map is a crucial factor affecting algorithm efficiency and coverage accuracy. To balance coverage accuracy and computational efficiency, this paper adopts a strategy based on the size of the agent: the resolution of each grid cell matches the coverage area of the agent, meaning each grid cell represents the area the agent can cover in one move. By setting an appropriate agent size, we ensure that the path planning precision is maintained while optimizing the use of computational resources.

The agent size directly influences the size of the grid cells and the resolution of the map. A smaller agent size means higher resolution, providing more detail, while a larger agent size means lower resolution, reducing memory usage. This method ensures that the grid map provides sufficient environmental details while optimizing memory usage and computational efficiency.

**Figure 5:**
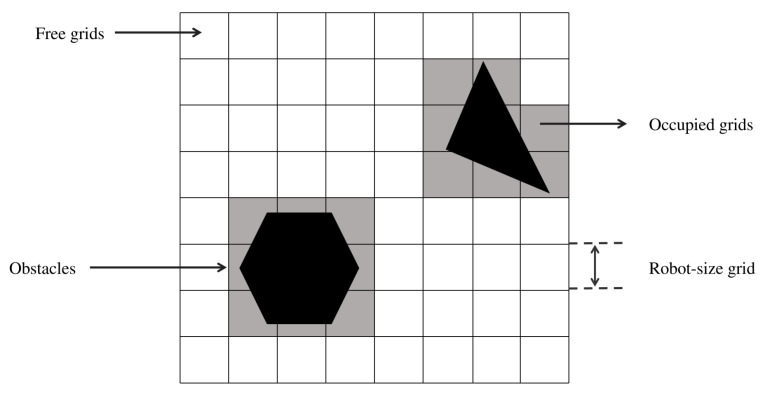
Environment segmentation into navigable and obstructed grids. The polygons and triangles represent actual obstacles, and the gray areas indicate the places that have already been occupied.

Before implementing the CCPP for the lawn mower robot, it is essential to observe the area that the robot needs to cover. In the grid map, the general area that the lawn mower robot needs to cover consists of all the blank grids. However, there are some special cases. For example, as shown in Figure 5, if a blank grid is surrounded by obstacles, the lawn mower robot cannot enter the blank grid for coverage.

**Figure 6:**
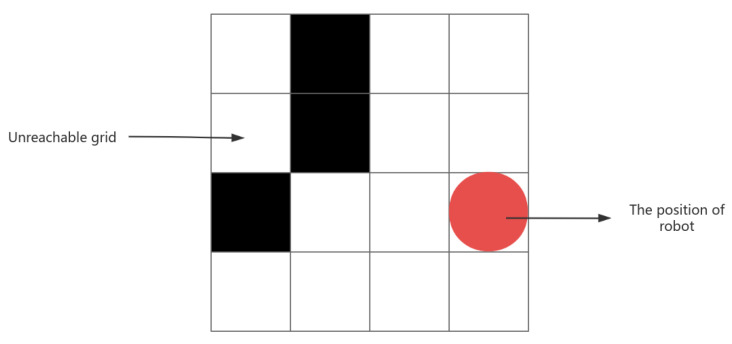
Unreachable grid.

Therefore, it is necessary to address this situation, whereby the obstructed blank grids on the map become grids with obstacles. This implies that if a grid cannot be covered due to the presence of obstacles around it, the grid will be considered to have obstacles, thus affecting the path planning process as shown in Figure 6.

**Figure 7:**
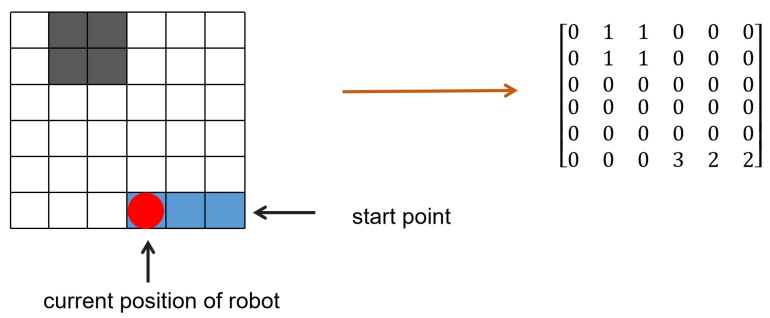
A state in the process. The left side shows the map, while the right side represents the state matrix in a grid mapping form.

For computers, maps are a series of two-dimensional matrix inputs. Firstly, it is necessary to convert the grid map into a two-dimensional state matrix form, where the elements representing empty grids are set to 0, the elements representing obstacle grids are set to 1, and the elements representing covered grids in the state matrix are also set to 1. When the agent appears randomly in the grid during training, its position is also marked as 1. The grid map is shown in Figure 7.

Because the perimeter of the map serves as the map boundary and is considered an obstacle, the lawn mower robot should not exceed the map boundary. Therefore, initially, add 1 around the two-dimensional matrix representing the grid map as shown in Equation (1):

(1) $$\left[\begin{array}{ccccc} 1.0 & 1.0 & 1.0 & 1.0 & 1.0\\ 1.0 & 0.0 & 0.0 & 0.0 & 1.0\\ 1.0 & 0.0 & 0.0 & 0.0 & 1.0\\ 1.0 & 1.0 & 1.0 & 1.0 & 1.0 \end{array}\right]$$

**Figure 8:**
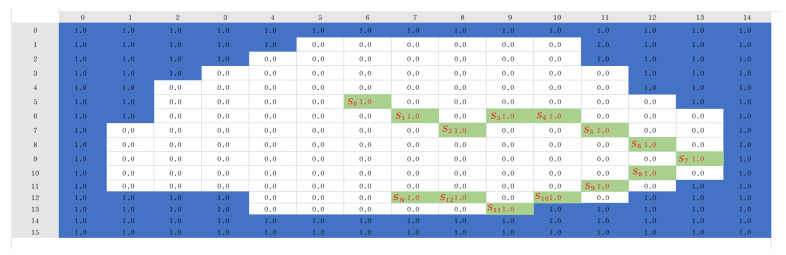
The random map for the *N*-th iteration.

In order to increase the randomness and diversity of the experiments [34], this study chooses to use newly generated maps for each episode as shown in Figure 8. The green squares in the figure represent the positions of dynamic obstacles, which move over time. The points marked as $S_{0}$, $S_{1}$,… indicate the positions of dynamic obstacles in different states. At different time points (states $S_{0}$, $S_{1}$, …, $S_{N}$), these dynamic obstacles may occupy different grid locations. The benefit of this approach is that the environment for each episode is randomly generated, which helps to evaluate the algorithm’s generalization ability and robustness. Additionally, using new maps can reduce the risk of over-fitting, as the algorithm does not overly adapt to specific maps but rather needs to adapt to different environments.

This approach is more akin to real-world scenarios, as robots often need to deal with various environments and situations. Furthermore, using a new map for each episode also helps to improve the reliability and reproducibility of the experiments, as the experimental results are not influenced by specific maps, making the results more convincing and credible.

**Figure 9:**
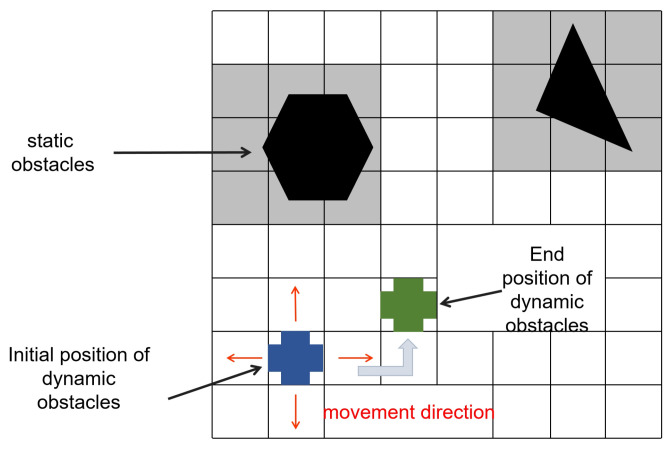
State representation.

Figure 9 shows a grid map containing both static and dynamic obstacles. The dynamic obstacle moves randomly in four different directions from its initial position. The green square represents the next state of the dynamic obstacle. The static obstacles (black shapes) remain stationary, while the dynamic obstacle (blue square) randomly moves to its destination (green square) in one of the directions indicated by the red arrows.

A sample state environment space is illustrated as in Figure 9, the environment in this paper is unknown to robots. The robots collect information in an unknown environment through continuous exploration. $S_{t}$ represents the position of the robots on the map. The action space of robots $\alpha_{t}=\left\{v_{t},L\right\}$, $v_{t}\in \left\{up,down,left,right\right\}.$ *L* is the duration of the robot’s movement. In many applications, the robot is required to move at a constant speed, so the *L* of each step of the robot is set as a fixed value.

## 3. Deep Reinforcement Learning Models and the Improvements

### 3.1. The DQN Algorithm

The state–action function gives the basis for RL algorithms to make decisions, either using existing info (exploitation) or trying new actions (exploration). In deep RL, a deep neural net approximates this function. The parameter update formula is

(2) $$\begin{array}{cc} Q(S_{t},A_{t},w) & \leftarrow Q\left(S_{t},A_{t},w\right)+\alpha \left[R_{t+1}+\gamma max_{a}\hat{q}\left(s_{t+1},a_{t},w\right)\right] \end{array}$$

In Equation (2), $Q(S_{t},A_{t},w)$ represents the Q-value when taking action $A_{t}$ in state $S_{t}$ based on the parameter *w*. α is the learning rate that controls the update magnitude. $R_{t+1}$ is the reward at time t+1. γ weighs the importance of future rewards. $max_{a}\hat{q}\left(s_{t+1},a_{t},w\right)$ is the maximum Q value among all actions in state $s_{t+1}$.

In DQN, the Q-value update method is based on the Q-learning algorithm [35] and incorporates neural networks to approximate the Q-value function. It minimizes the mean squared error between the target Q value and the current Q value; finally, through updates of the target network and the main network, DQN can stably update the Q-value function, thereby learning the optimal policy:

(3) $$\begin{array}{c} \Delta w=\alpha \left(R_{t+1}+\gamma max_{\alpha }\hat{q}\left(s_{t+1},a_{t},w\right)-\hat{q}\left(s_{t},s_{t},w\right)\right)\cdot \nabla_{w}\hat{q}\left(s_{t},a_{t},w\right) \end{array}$$

In Equation (3), Δw represents the update amount of the parameters. $\nabla_{w}\hat{q}\left(s_{t},a_{t},w\right)$ is the gradient of the Q-value function with respect to the parameter *w* and is used to determine the update direction.

**Figure 10:**
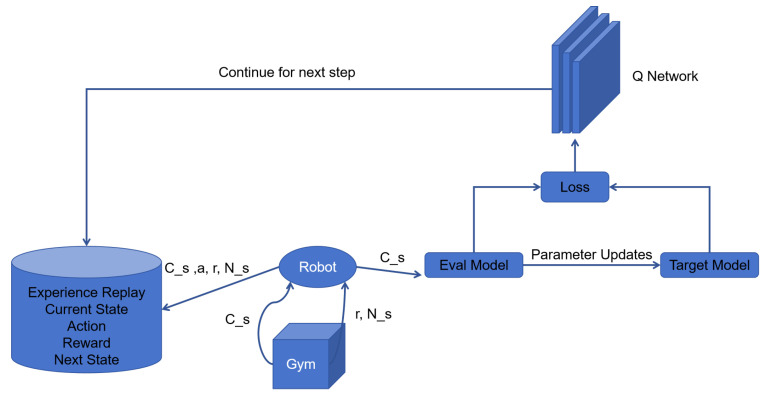
The framework of DQN.

The DQN architecture has two neural nets, the Q network and the target network [36] and a component called the experience replay as shown in Figure 10. A deep neural network Q-network is used to approximate the state–action function [37]. When the agent interacts with the environment, each experience is stored in a replay buffer and later randomly sampled to train the Q network. The DQN is trained over many episodes with multiple steps, undergoing a series of operations in each time step.

First, the parameters of the Q network and the target network are randomly initialized. During the interaction with the environment, the agent collects experience data, and then selects actions using the ε-greedy policy. The experience replay executes the greedy action and receives the next state and reward [38]. It saves this observation as a sample of the training data and inputs it into both networks.

**Figure 11:**
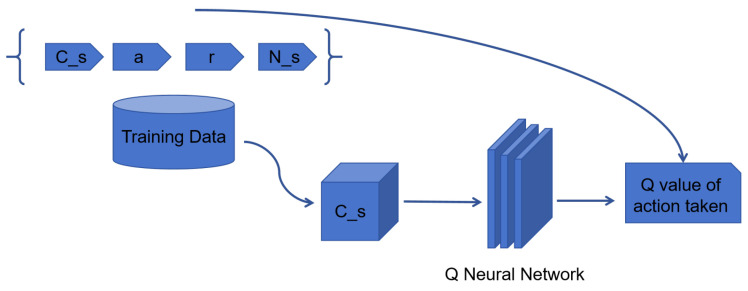
Q network predicts Q value.

The Q network takes the current state and action from each data sample and predicts the Q value for that particular action as shown in Figure 11. This is the ‘predicted Q value’.

The target network evaluates all possible actions for the next state and selects the action with the highest Q value to obtain the “best Q value” for that state. This best Q value is then multiplied by the discount factor γ to obtain the target Q value.

After training the DQN, the mean squared error loss is computed using the difference between the target Q value and the predicted Q value. Then, the loss is back-propagated to update the weights. After T steps of updating the target network and the main network, it can predict more accurate Q values.

### 3.2. The Re-DQN Algorithm

This paper proposes an improved DQN algorithm, called Re-DQN, to enhance the efficiency and stability of path planning. Complex terrain features are introduced in the environment design to simulate various obstacles and terrain variations. To strengthen the overall relevance of the model, a replay buffer is used to update the action values in each iteration, ultimately guiding the model’s training and optimizing the decision-making process. The Re-DQN algorithm is as shown in Algorithm 1.
**Algorithm** **1** Re-DQN
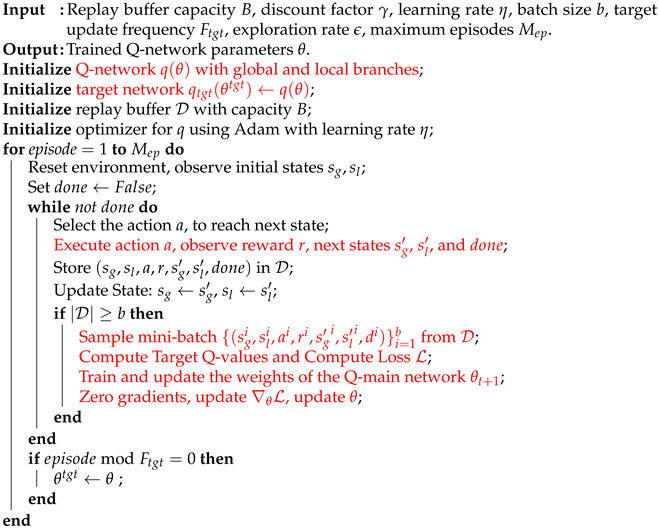


#### 3.2.1. Improvements Based on Action Selection

In complete coverage path planning, the robot selects appropriate actions in different states to achieve efficient coverage. To effectively balance exploration and exploitation, this study divides action selection into two parts: a dynamic ε-greedy strategy [39] combined with curiosity-driven exploration and a soft-max strategy [40].

The dynamic ε-greedy strategy and curiosity-driven exploration mechanism are used in combination. The core of the dynamic ε-greedy strategy lies in the gradual decay of the ε value. Initially, ε is set to a high value to encourage the robot to explore more. As training progresses, the ε value gradually decreases, prompting the robot to rely more on the learned strategy. The specific formula is

(4) $$\varepsilon =\varepsilon_{\mathrm{end}}+\left(\varepsilon_{\mathrm{start}}-\varepsilon_{\mathrm{end}}\right)\times \exp\left(-\frac{S_{\mathrm{done}}}{\varepsilon_{\mathrm{decay}}}\right)$$

In Equation (4), $S_{\mathrm{done}}$ measures the training progress. At each step, a random number *r* is generated and compared to the current ε value. If r>ε, then the action with the highest current evaluation value is selected; otherwise, an action is chosen at random.

Building on this, a random noise proportional to ε is added to the state evaluation values as a curiosity bonus. This encourages the robot to explore states with higher uncertainty or novelty, preventing it from getting stuck in local optima and gradually shifting towards exploiting the learned strategy over time. This improves the efficiency and effectiveness of the complete coverage path planning.

Alongside the ε-greedy strategy, a soft-max strategy is also used, converting state evaluation values into a probability distribution for action sampling. Instead of relying on simple evaluation values, intrinsic curiosity-driven rewards are combined to enhance action selection. The curiosity bonus is added to the state evaluation values before applying the soft-max function. It can be computed based on the novelty of the state (such as visit frequency) or prediction error, encouraging the robot to explore novel and uncertain areas more effectively.

The action selection is then performed using the soft-max function:

(5) $$q_{i}=\frac{exp(z_{i})/T}{\Sigma_{j}exp\left(z_{j}/T\right)}$$

where $z_{i}$ represents the evaluation value of action *i*, and T is the temperature coefficient.

In addition to the ε-greedy strategy, we also use the soft-max strategy, which converts state evaluation values into a probability distribution through the soft-max function, from which actions are sampled. A temperature coefficient T is introduced to control the smoothness and diversity of the action selection [41,42]. The use of the temperature coefficient ensures more diverse action selection in the early stages of exploration, gradually converging to the optimal policy as training progresses. Typically, the soft-max strategy is used in scenarios that require more nuanced probability control, and the formula is as follows.

#### 3.2.2. Reward Function

In CCPP, designing an appropriate reward function is crucial, as it guides the agent to learn suitable behaviors. It encourages the agent to cover uncovered areas quickly while minimizing redundant coverage of already covered regions [43]. Designing the reward function is a critical step, as it directly impacts the learning efficacy and task execution efficiency of the agent.

To calculate intrinsic rewards based on state novelty, it is suggested to use an exponential decay function based on visit frequency. This definition effectively encourages the robot to prioritize exploring unvisited or rarely visited areas while quickly reducing the reward after a state is repeatedly visited, thus avoiding the waste of exploration resources. The following is the definition of the intrinsic reward using an exponential decay function based on the state novelty:

(6) $$r_{\mathrm{intrinsic}}\left(s\right)=e^{-N(s)}$$

where N(s) represents the visit frequency of state *s*, and the reward decays exponentially as the state is visited more frequently.

The reward function is defined as follows:

(7) $$r=\left\{\begin{array}{cc} -P_{\mathrm{move}} & \\ r-P_{\mathrm{obstacle}} & \mathrm{if}\;\mathrm{collision}=1\\ r+R_{\mathrm{discover}}\times N_{\text{covered\_tiles}} & \mathrm{if}\;N_{\text{covered\_tiles}}=1\;\mathrm{and}\;\mathrm{collision}=0\\ r+\left(\frac{N_{\text{covered\_1}}}{N_{\text{covered\_0}}+N_{\text{covered\_0}}}\right)\times P_{\mathrm{move}} & \mathrm{if}\;N_{\text{covered\_tiles}}=1\;\mathrm{and}\;\mathrm{collision}=0\\ r-P_{\mathrm{move}} & \mathrm{if}\;N_{\text{covered\_tiles}}=0\;\mathrm{and}\;\mathrm{collision}=0\\ r+R_{\mathrm{cc}} & \mathrm{if}\;C=1\\ r-P_{\mathrm{terrain}}\times \max\left(0,T_{\mathrm{diff}}\right) & \mathrm{if}\;T_{\mathrm{info}}=1\\ r+\lambda \cdot r_{\mathrm{intrinsic}} & if\;N(s)\geq 0 \end{array}\right.$$

In the equation above, *r* is the reward, and $P_{\mathrm{move}}$ is a movement penalty for each step. In this way, the agent incurs a penalty for each step it takes, thereby encouraging it to choose shorter paths [44]. This approach ensures that the path length is effectively considered, rather than solely focusing on the destination of the path. *C* stands for “complete coverage completion”. When *C* is 1, it means the agent has successfully covered all the accessible areas, and the task is complete. $T_{\mathrm{info}}$ = 1 indicates that terrain information is present. $T_{\mathrm{diff}}$ represents the terrain difference between the current position and the target position. $P_{\mathrm{obstacle}}$ is a collision penalty, and $R_{\mathrm{discover}}$ is a reward for exploring new areas. $R_{\mathrm{CC}}$ is the reward given by the agent when they complete the map, and $P_{\mathrm{terrain}}$ is a penalty for terrain differences, while $N_{\text{covered\_tiles}}$ is the number of areas that are newly covered by the agent.

#### 3.2.3. Improved DQN Network Architecture

In path planning using DQN [45,46], the number of obstacles in the environment is dynamically changing, which causes the input dimensionality to fluctuate. This, in turn, affects the stability of the DQN model. To overcome this challenge, a dynamic input structure is designed, which can adapt to changes in the number of obstacles in the environment while ensuring that the DQN model always processes input data with a fixed dimensionality during both training and inference.

Assuming that the system can handle a maximum of *n* obstacles, and the number of obstacles in the environment is *m*, the composition of the input vector input depends on the number of obstacles. Below is a piecewise function that expresses how the input structure is processed, clearly outlining how the input dimensionality is handled under different obstacle counts:

(8) $$\mathrm{input}\left(m\right)=\left\{\begin{array}{cc} {}[o_{a},o_{t},o_{1},o_{2},...,...,...,...,o_{n}], & \mathrm{if}\;m\geq n\\ {}[o_{a},o_{t},o_{1},o_{2},...,o_{m},0,0,....,0], & \mathrm{if}\;m<n \end{array}\right.$$

The explanation is as follows:

- $o_{a}$ represents the state of the robot ($x,y,z,v_{x},v_{y},v_{z}$).

- $o_{t}$ represents the state of the target (x,y,z).

- $o_{1},o_{2},o_{3},\ldots \ldots o_{n}$ represent the states of the first *n* obstacles ($x,y,z,v_{x},v_{y},v_{z}$).

When m=n, the environment has exactly the maximum number of obstacles that our system can handle. In this case, the input vector $\mathrm{input}\left(m\right)=\left[o_{a},o_{t},o_{1},o_{2},...,o_{n}\right]$. When m>n, we have more obstacles in the environment than our system is designed to handle comprehensively. In this situation, we choose to still use the same input vector.

Zero Vector **0**: When the number of obstacles *m* in the environment is less than the maximum number of obstacles *n* set by the system, the zero vector **0** is used to fill the empty positions corresponding to the obstacle states in the input vector to ensure that the dimension of the input vector is always a fixed length related to *n*.

To improve the fitting of Q values, an incentive layer is added between the hidden layer and the output layer. This layer applies an incentive value to different Q values, enhancing the accuracy and effectiveness of the Q-value estimation. This allows the model to better adapt to environmental changes, thereby accelerating the training process and improving the model’s performance.

In standard DQN, the Q-value update formula is

(9) $$Q\left(s,a\right)=Q\left(s,a\right)+\alpha \left[r+\gamma \max_{a^{\prime }}Q\left(s^{\prime },a^{\prime }\right)-Q\left(s,a\right)\right]$$

To encourage the agent to avoid obstacles, the dynamic incentive value can be adjusted based on the following two factors:

- Proximity: This function calculates the distance between the current action and the obstacles. The closer the action is to an obstacle, the lower the dynamic incentive value (i.e. negative incentive):

(10) $$\mathrm{Proximity}\left(s,a\right)=-\frac{1}{d(s,a)}$$

where d(s,a) is the distance between the agent and the nearest obstacle in the new state $s^{\prime }$ after performing action *a*.

- Obstacl_Density: This function measures the density of obstacles in the environment. The higher the obstacle density, the more negative the incentive applied by the system, encouraging the agent to avoid areas with dense obstacles:

(11) $$\text{Obstacl\_Density}\left(s\right)=\frac{\mathrm{Number}\;\mathrm{of}\;\mathrm{obstacles}\;\mathrm{in}\;\mathrm{region}(s)}{\mathrm{Area}\;\mathrm{of}\;\mathrm{region}(s)}$$

The formula for the dynamic incentive value Dynamic_Incentive(s,a) can be expressed as:

(12) $$\text{Dynamic\_Incentive}\left(s,a\right)=\lambda_{1}\cdot \mathrm{Proximity}\left(s,a\right)+\lambda_{2}\cdot \text{Obstacl\_Density}\left(s\right)$$

where:

- $\lambda_{1}$ and $\lambda_{2}$ are hyperparameters used to adjust the importance weights of Proximity and Obstacle_Density, respectively.

To further enhance exploration capabilities, a noisy-linear layer [47] is introduced, which injects random perturbations into the weights and biases of the network, improving the exploratory behavior of the model and the stability of training. Experiments demonstrate that the improved DQN significantly enhances the coverage rate and path planning efficiency, while also exhibiting stronger robustness and generalization in various environments. This design effectively leverages the complementarity of the global and local information, making the model better suited for complex and dynamic environments.

In the noisy-linear layer, we introduce noise to perturb the weights and biases. The formula is

(13) $$\begin{array}{cc} & W_{\mathrm{noisy}}=W_{\mu }+W_{\sigma }⊙\epsilon_{W}\\ & b_{\mathrm{noisy}}=b_{\mu }+b_{\sigma }⊙\epsilon_{b} \end{array}$$

During each forward pass, the noise parameters are regenerated. The specific forward pass formula is

(14) $$y=\left(\begin{array}{c} W_{\mu }+W_{\sigma }⊙\epsilon_{W} \end{array}\right)x+\left(\begin{array}{c} b_{\mu }+b_{\sigma }⊙\epsilon_{b} \end{array}\right)$$

#### 3.2.4. Environmental Terrain Design

This study employs noise generation methods to create diverse and realistic terrain features. Due to the noise characteristics of the generator, the terrain maps may exhibit some random textures or fluctuations, represented by different grayscale regions. The terrain generation process includes the following steps:

(1) Noise Generation: The noise generator generates noise maps with specified dimensions and frequency. These noise maps serve as the foundation for creating terrain features.
(2) Normalization: The generated noise maps are normalized to ensure that the terrain values range from 0 (representing the lowest altitude) to 1 (representing the highest altitude). This normalization helps to evenly represent different terrain heights.

Perlin noise is a gradient noise function used to generate natural textures, and it is widely employed in computer graphics to create textures, terrains, and more. The generation of Perlin noise involves several steps, including gradient calculation and interpolation. Here are the basic formulas for Perlin noise:

(15) $$\begin{array}{cc} \mathrm{PN}(x,y) & =\mathrm{lerp}\left(\mathrm{lerp}\left(\mathrm{dot}(i,j),\mathrm{dot}(i+1,j),s(x)\right),\right.\\ \; & \;\mathrm{lerp}\left(\mathrm{dot}(i,j+1),\mathrm{dot}(i+1,j+1),s(x)\right),\left.s(y)\right) \end{array}$$

where we have the following:

(1) lerp(a,b,t) represents a linear interpolation function: lerp(a,b,t)=a+t×(b−a).
(2) s(x) is the smooth interpolation function, typically using the cubic Hermite function:

(16) $$s\left(x\right)=3x^{2}-2x^{3}.$$

Perlin noise is often created by combining multiple layers of noise with different frequencies and amplitudes to achieve a more complex effect. This process is known as “Octaves”:

(17) $$\mathrm{PNO}\left(x,y\right)=\sum_{k=0}^{n-1}\left(\frac{1}{2^{k}}\times \mathrm{PN}\left(\frac{x}{2^{k}},\frac{y}{2^{k}}\right)\right)$$

where *n* represents the number of layers (octaves).

In the experiment, we configure the terrain generator to generate terrain maps with varying sizes and frequencies. Specifically, we can set the map dimensions and adjust the frequency parameters to evaluate the impact of different levels of detail on the generated terrain.

**Figure 12:**
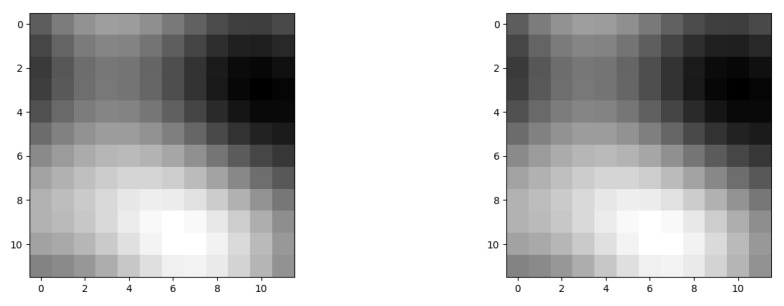
Ordinary-terrain map.

**Figure 13:**
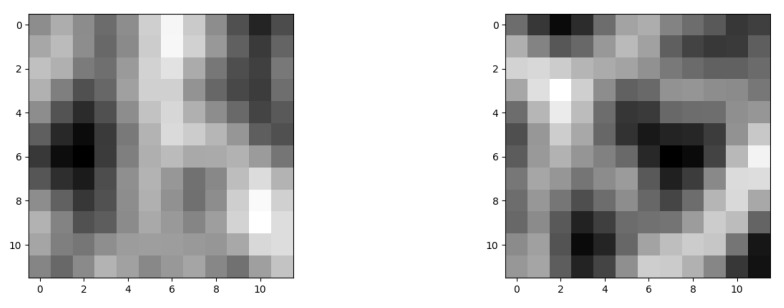
A map with a more complex terrain.

As shown in Figure 12 and Figure 13, grayscale values are used to represent the elevation of the terrain. Darker areas generally indicate lower terrain, while lighter areas indicate higher terrain. These grayscale values have been normalized to be displayed within an appropriate range in the images.

## 4. Simulation Results and Discussion

### 4.1. Setting of Simulation Conditions

To research and optimize the performance of the improved DQN algorithm for CCPP, simulations of the mobile robot’s path planning on environmental maps have been conducted. The experimental objectives include the following:

(1) Validate the effectiveness of the improved DQN algorithm, i.e., verify the path coverage capability of the improved DQN in different environments through experiments, and assess its adaptability and performance in various complex scenarios.
(2) Evaluate the impact of key hyperparameters during training, that is, adjust hyperparameters such as the exploration rate, discount factor, and learning rate to analyze their effects on model training performance and stability.
(3) Enhance the model’s adaptability in complex environments, i.e., test the model in environments with obstacles or irregular boundaries, study the performance of the DQN algorithm in such environments, and propose corresponding optimization strategies.

During the simulation experiment process, the basic environment parameters, rewards, and Re-DQN network model parameters are initialized first. Then, a preprocessed grid map, obstacle map, and coverage map are constructed, with the obstacle frequency, fill ratio, and height frequency set within the obstacle map.

Each time training begins, the lawnmower robot’s position randomly appears on the grid map, and obstacles within the grid map change randomly. It is ensured that the lawnmower robot’s initial position is not obstructed by obstacles. Subsequently, at each time step, the agent selects the next action based on the current state and path planning algorithm, updates the agent’s position and map, and computes the immediate reward.

Termination conditions include the following:

(1) Coverage map indicating all reachable areas have been visited.
(2) Agent collides with an obstacle.
(3) Agent reaches the boundary of the map.

In summary, condition (1) represents a successful termination condition, while condition (2) represents a failure termination condition.

During training, each action generates a sample, stored in a replay memory pool.

In the CCPP based on deep reinforcement learning, my evaluation metrics mainly include three aspects: the average total nb_step, which measures the average number of actions taken by the agent to complete the task; the average total tiles_visited, which reflects the number of different areas covered by the agent during task execution; and the average total reward, which assesses the average feedback received by the agent during task execution. These metrics collectively evaluate the effectiveness of the path planning and the performance of the agent.

The simulation experiments in this study are run in an Ubuntu 20.04 environment, using Python as the development language. The parameters for the simulation platform are listed in Table 1.

### 4.2. Outdoor Map Simulation Experiment

**Figure 14:**
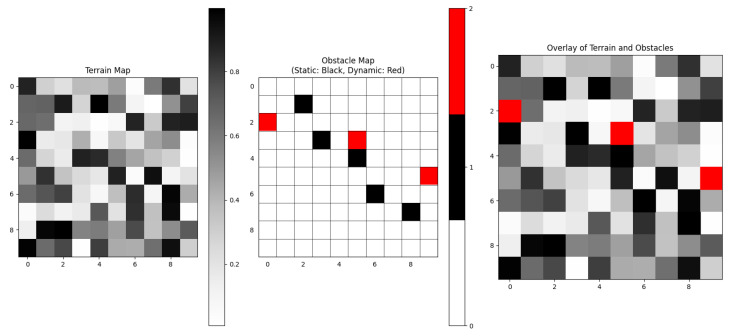
Overlay of terrain and obstacle distribution (10 × 10).The first figure is the terrain map, and the second figure is the obstacle map, where the black squares represent static obstacles and the red squares represent dynamic obstacles. The third figure shows the overlap of the terrain map and the obstacle map.

Figure 14 consists of three parts: the left side shows the terrain map; the middle displays the obstacle map, with static obstacles in black and dynamic obstacles in red; the right side shows the overlay of the terrain and obstacle maps, combining terrain height information with obstacle locations, illustrating the spatial relationship between the terrain and the obstacles.

We observe how agents navigate different types of terrain and how the complexity of the terrain affects their performance. The terrain influences the path planning methods in the following ways:

(1) Movement cost differences: Varying terrain heights result in different movement costs for agents, with the algorithm favoring paths with lower costs.
(2) Accessibility: Areas with significant elevation differences may be considered impassable, requiring the path planning algorithm to avoid these regions.
(3) Reward mechanism: The terrain information is integrated into the reward function, where larger elevation changes incur penalties, encouraging agents to select flatter paths.
(4) Environmental complexity: The terrain adds complexity to the planning process, requiring the algorithm to balance terrain difficulty with coverage efficiency.

Overall, the terrain affects the cost, accessibility, and efficiency of path planning. Preliminary results indicate that terrain maps with higher levels of detail provide a more challenging environment for the agents, thereby influencing their navigation and decision-making processes.

### 4.3. Parameter Analysis

First, set the size of the agent, ensuring that the size is greater than or equal to 1. Next, configure the agent’s field of view and rotation capabilities. Before conducting experiments, it is essential to perform a validity check on the field of view. The field of view may not be set, in which case the agent might not have any vision constraints. If the field of view is to be set, it must be a positive odd integer greater than or equal to 1. This ensures that the field of view is symmetrically distributed around the agent, facilitating calculations and processing.

In this experiment, the settings for the modified DQN parameters need to be carefully considered in light of the environment, task nature, and resource constraints. Below is a detailed explanation and setting recommendations for each parameter:

(1) *N*: Determines the number of samples stored in the experience replay buffer. A buffer that is too small may lead to less diverse samples, affecting the model’s generalization ability, while a buffer that is too large may increase memory demands. It is generally set between 5000 and 100,000. For simpler tasks, 5000 may be sufficient. For more complex tasks or if ample memory resources are available, a larger size can be chosen.
(2) γ: A larger discount factor means the model focuses more on long-term rewards, while a smaller factor means the model focuses more on short-term rewards. It is usually set between 0.9 and 0.99. A value of 0.9 indicates that the importance of future rewards gradually decreases, suitable for short-term decision tasks; 0.99 is more appropriate for tasks with a longer time span.
(3) ϵ: Initially, a high exploration rate helps in exploring new strategies; as training progresses, the exploration rate gradually decreases, leading to more reliance on the learned strategy.
  - $\epsilon_{\mathrm{start}}$: 0.9 to 1.0. Usually set high to encourage more exploration at the beginning.
  - $\epsilon_{\mathrm{end}}$: 0.01 to 0.1. A lower value ensures that the model relies more on the learned strategy during the later stages of training.
  - $\epsilon_{\mathrm{decay}}$: 2000 to 10,000. A higher decay value means a longer exploration period, suitable for more complex tasks.
(4) τ: Frequency of updating the target network. A lower update frequency may lead to delayed target updates, while a higher frequency may cause instability in training. It is recommended to set it between 500 and 5000 steps. For simpler tasks, 500 steps may suffice; for more complex tasks, a higher step count can be selected to stabilize training.
(5) α: Controls the step size of each parameter update. A learning rate that is too high may cause instability in training, while a learning rate that is too low may result in slow or stagnant training. It is usually advisable to start with a small learning rate, such as 0.001, and adjust based on the training outcomes.
(6) Environment and reward parameters: Additional settings related to the environment and rewards, which are not extensively discussed here, can be adjusted based on the size of your map and the terrain you wish to design. Relevant parameters include the following:
  - $P_{\mathrm{move}}$: 0.01–0.1.
  - $P_{\mathrm{terrain}}$: 0.01–0.1.
  - $P_{\mathrm{obstacle}}$: 0.1–1.0: adjusted according to the density of obstacles and the agent’s ability to avoid them. A higher value should be set if you want the agent to be very sensitive to obstacles.
  - $P_{\mathrm{discover}}$ and $P_{\mathrm{coverage}}$: If the task focuses on coverage and discovering new areas, increase the values of $P_{\mathrm{discover}}$ and $P_{\mathrm{coverage}}$. If the task emphasizes precise and efficient path planning, consider increasing $P_{\mathrm{move}}$ and $P_{\mathrm{obstacle}}$ values.

### 4.4. Complete Coverage Path Planning Results

**Figure 15:**
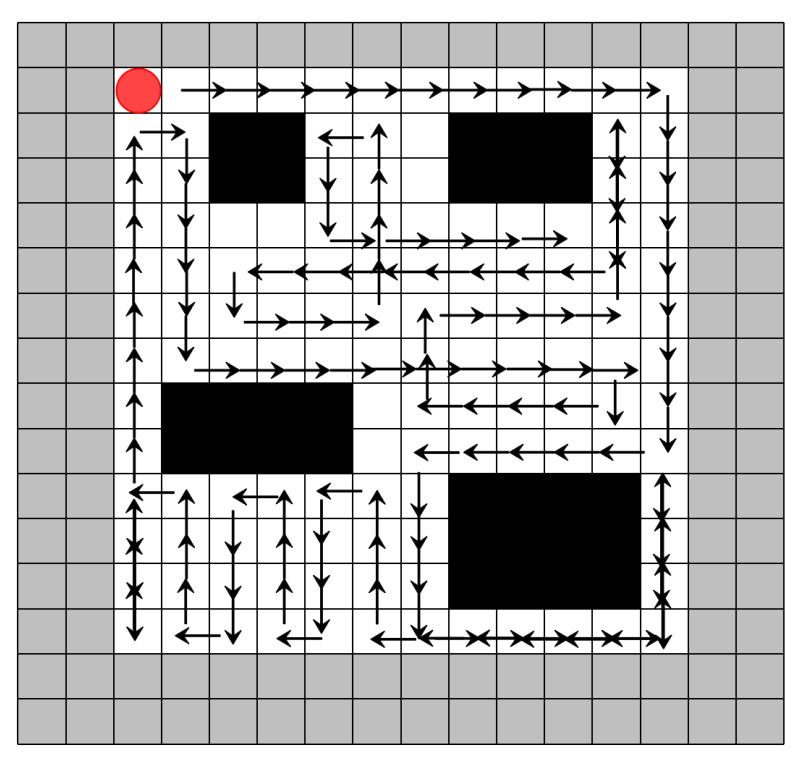
Coverage trajectory with DQN-based CCPP (16 × 16). The red circle represents the starting position, the gray border indicates the boundary of the map, and the mowing robot should not go beyond the map boundary. Black squares represent obstacles, and the arrows indicate the direction of movement.

**Figure 16:**
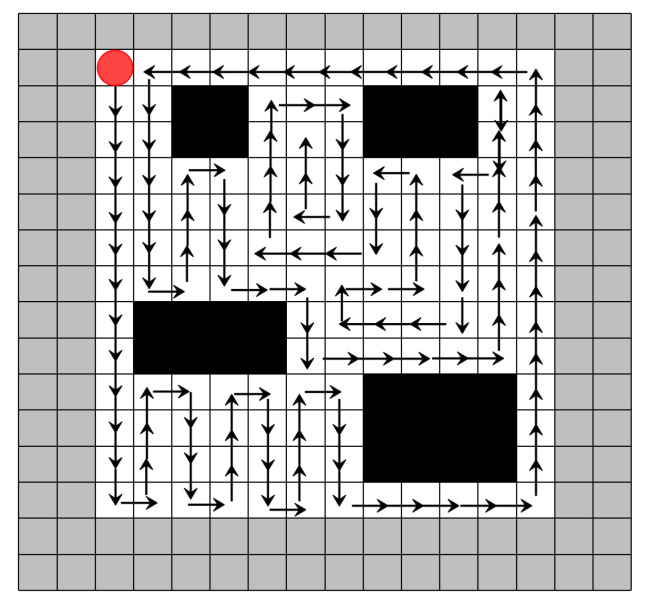
Coverage trajectory with Re-DQN-based CCPP (16 × 16). The red circle represents the starting position, Black squares represent obstacles, and the arrows indicate the direction of movement.

First, a detailed comparison of the CCPP trajectories is conducted. In the first scenario, with a grid size of 16 × 16, the CPP trajectories for the simplest DQN-based algorithm, the improved DQN, are shown in Figure 15 and Figure 16, respectively. It is evident that our algorithm has a clear advantage in CCPP compared to standard DQN.

**Figure 17:**
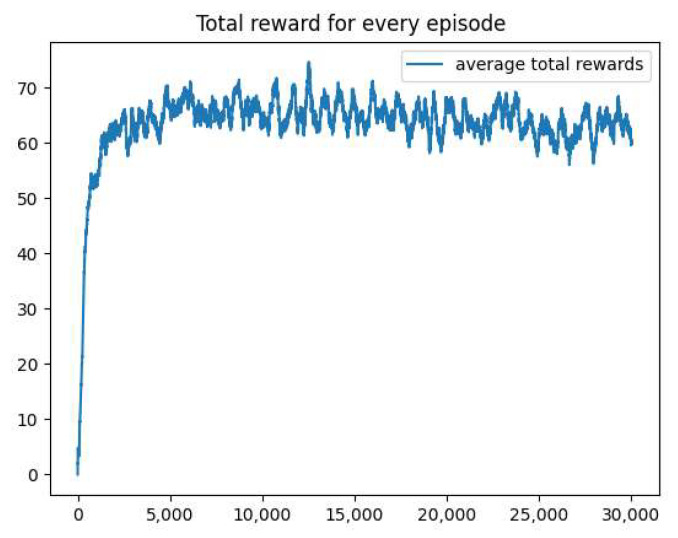
The Q-learning reward. The reward of Q-learning increases rapidly in the early stage, then fluctuates. The training process is unstable.

Figure 17 shows the total reward variation across episodes during training in the CCPP task based on the Q-learning algorithm. The horizontal axis represents the number of training episodes, and the vertical axis represents the average total reward per episode.

As shown in Figure 17, the reward of the Q-learning algorithm increases rapidly within the first 5000 episodes, but there are still significant fluctuations after reaching a certain level. This indicates that the model may exhibit some instability during the training process.

Additionally, the reward reaches a relatively high level in the early stages (around 3000 episodes) and does not show significant improvement afterward. This may indicate that the model’s learning ability is limited and unable to further optimize its strategy, demonstrating the limited performance of Q-learning in the complex task of CCPP.

In path planning or coverage tasks, nb_step represents the number of steps the agent takes to complete the task. avg_nb_steps is the average number of steps over a recent period, reflecting the efficiency of the agent in executing the task.

If the value of avg_nb_steps decreases during training, it indicates that the agent is completing the task with fewer steps, which means it is becoming more efficient. The decreasing avg_nb_steps during training typically suggests that the agent’s strategy is improving over time.

**Figure 18:**
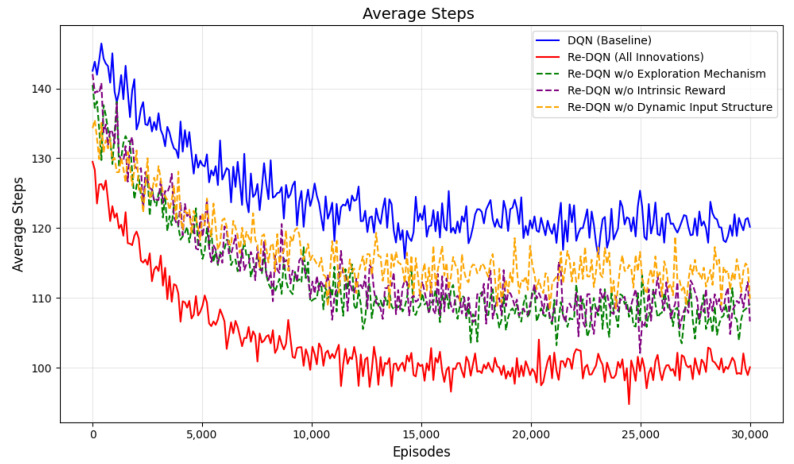
Average steps. It shows the variation in the average steps of different algorithms during the training process.

In Figure 18, it can be seen that DQN typically requires more steps, around 120, to complete the task throughout the training process. Re-DQN generally requires fewer steps in the same number of episodes, eventually stabilizing at around 100 steps. This indicates that Re-DQN performs better on this task as demonstrated by its lower average number of steps, and validates the importance of each component in improving the strategy.

In coverage tasks, avg_tiles_visited represents the average number of tiles covered by the agent in each episode. For coverage tasks, the goal is typically for the agent to cover as much of the area as possible. Therefore, the larger the avg_tiles_visited, the more extensive the area covered by the agent, indicating higher coverage efficiency. This suggests that the agent is continuously improving its strategy and is better able to complete the coverage task.

**Figure 19:**
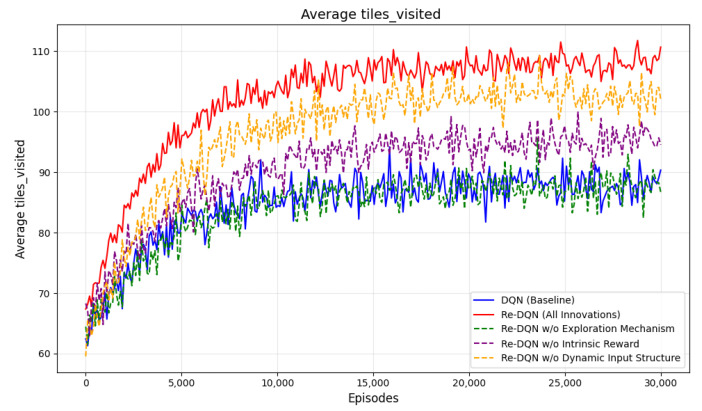
Average tiles_visited. It shows the changes in the average tiles visited of different algorithms during the training process.

Figure 19 shows the average number of tiles covered by different algorithms in each episode in a CCPP task. Re-DQN covers more tiles per episode, around 108 tiles, while DQN covers about 87 tiles. This indicates that Re-DQN is more efficient in exploration and coverage, allowing it to cover a larger area in the same amount of time.

**Figure 20:**
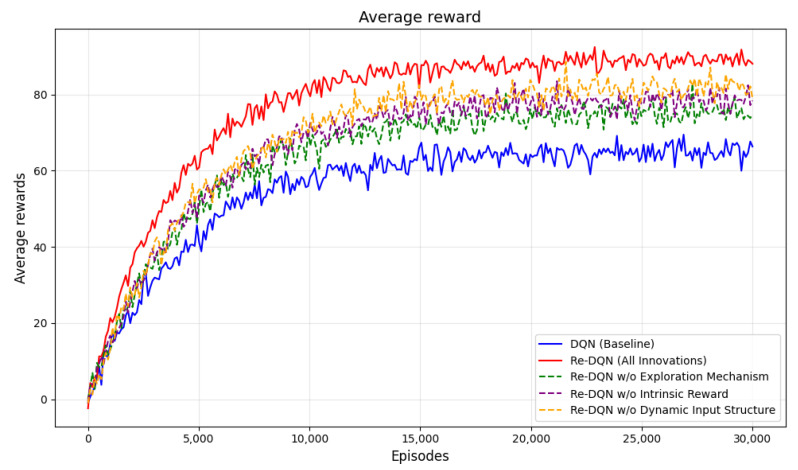
Average reward. It shows the changes in the average rewards of different algorithms, fully indicating that the Re-DQN algorithm performs better in this task.

Figure 20 illustrates the different performance of the models during the training process. The Re-DQN model stabilizes at a higher total reward level, around 85, indicating superior performance compared to DQN, which stabilizes at around 65.

In terms of convergence speed, the Re-DQN model improves rapidly, with total reward levels increasing rapidly, demonstrating faster learning capability. The DQN model, however, has a slower overall improvement speed, and in the later stages, exhibits significant fluctuations, suggesting that the model’s strategy has not yet fully stabilized.

In conclusion, Table 2 shows the comparison of different algorithms in the task, indicating that removing certain innovations may lead to a decrease in performance. On the other hand, Re-DQN achieves the best performance through its comprehensive optimization strategy.

**Figure 21:**
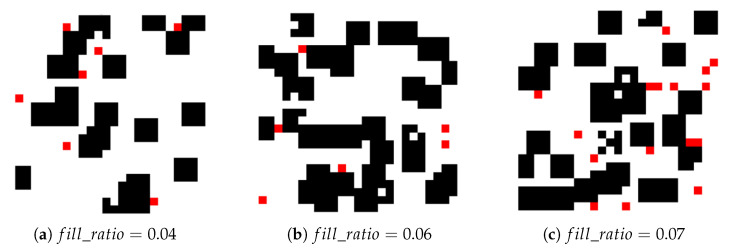
Obstacle maps with different fill ratios. It shows three obstacle scenes with different fill ratios. Each scene contains black squares (static obstacles) and red squares (dynamic obstacles). With different fill ratios, the scenes and the number of obstacles are different.

Figure 21 displays obstacle maps under different fill ratios. The (a)–(c) represent the obstacle layouts for different fill ratios (0.04, 0.06, and 0.07).

From Table 3, we can see that the performance of each algorithm varies under different obstacle fill ratios. Re-DQN consistently performs as the strongest algorithm in all tests, being able to adapt to environments with higher obstacle densities, maintaining a coverage rate ranging from 94% to 100%.

**Figure 22:**
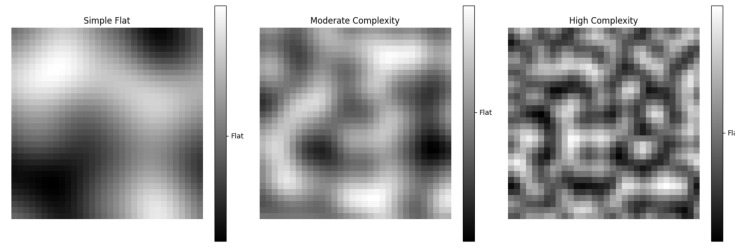
Varying terrain complexity. It shows three terrain images with different levels of complexity. The gradient bar displays color changes from light to dark. Larger color changes indicate more complex terrain.

Figure 22 depicts varying levels of terrain complexity, including simple flat, moderately complex, and highly complex environments, to evaluate the algorithm’s performance under different conditions.

Based on Table 4, we can conclude that in complex environments, Re-DQN, compared to the other path planning algorithms, demonstrates higher flexibility and robustness, making it more suitable for more complex and diverse geographical environments.

Table 5 systematically compares the performance of traditional algorithms and reinforcement learning algorithms in coverage tasks through multiple metrics, including path length, coverage rate, and path redundancy rate. Re-DQN demonstrates its superiority with shorter paths, lower redundancy rates, and higher adaptability scores, while maintaining moderate complexity. This highlights Re-DQN as an efficient and balanced solution for coverage tasks.

## 5. Conclusions

### 5.1. Main Conclusions and Findings

This study proposed a novel algorithm, Re-DQN, for complete coverage path planning in lawn mowing robots using deep reinforcement learning. Taking into account the limitations of traditional algorithms, Re-DQN improved tiles_visited by 24.14%, increased the reward level by approximately 30.76%, and increased step efficiency by 16.67%.

By modeling the environment and dynamically adjusting the grid map resolution based on the robot’s size, the algorithm introduces a new exploration mechanism for action selection, significantly enhancing the robot’s ability to explore new areas and optimize paths, thus effectively avoiding local optima. A dynamic input structure is incorporated to address the challenges posed by changes in the number of obstacles in the environment. This improvement enables the robot to better navigate high-dimensional continuous state spaces, ensuring more comprehensive coverage and reducing planning time. The carefully designed reward function encourages the robot to cover new areas while minimizing redundant coverage, penalties for collisions, and terrain differences, leading to more efficient path planning.

### 5.2. Main Limitation of the Research

In the modeling phase, the actual motion characteristics of the lawn mower robot were not fully considered. The real-world model is much more complex than the one used in this study. Therefore, this complete coverage path planning algorithm may perform poorly in certain situations. Despite optimization of the exploration strategy, the algorithm may still fall into local optima, especially in large-scale or diverse environments. Finally, while the current algorithm performs well in single-robot systems, its scalability and coordination in multi-robot systems require further research and optimization.

### 5.3. Future Research Prospects

To further enhance its practical value and performance [48], future work will focus on studying the adaptability of Re-DQN in complex environments. This will involve testing and optimizing the Re-DQN algorithm in more complex and dynamically changing environments, including handling different types of environmental conditions. Additionally, we will explore the potential of extending the Re-DQN algorithm to multi-robot systems, investigating collaboration strategies and communication mechanisms between robots to achieve more efficient and large-scale complete coverage path planning.

## Figures and Tables

**Table 1:** Simulation platform.

OS	Language	CPU	GPU	RAM
Ubuntu 22.04	Python 3.8	Intel i5-13400	RTX 4070 Ti	12 Gb

**Table 2:** Algorithm comparison.

	DQN	Re-DQN	Re-DQN w/o EM	Re-DQN w/o IR	Re-DQN w/o DIS
Steps	120	100	108	110	115
Tiles visited	87	108	88	96	101
Rewards	65	85	72	79	81

**Table 3:** Algorithm coverage efficiency under different fill_ratio.

Algorithm	*fill_ratio* = 0.04	*fill_ratio* = 0.06	*fill_ratio* = 0.07
Boustrophedon	92%	89%	86%
A* Coverage Algorithm	95%	94%	93%
DQN	87%	83%	78%
DDQN	89%	83%	82%
Dueling DQN	87%	84%	81%
PPO	95%	92%	90%
Re-DQN (Our algorithm)	100%	97%	94%

**Table 4:** Algorithm coverage efficiency under different terrain complexities.

Algorithm	Simple Flat	Moderate Complexity	High Complexity
Boustrophedon	92%	83%	78%
A* Coverage Algorithm	96%	91%	83%
DQN	84%	83%	78%
DDQN	86%	83%	80%
Dueling DQN	87%	84%	81%
PPO	95%	92%	86%
Re-DQN (Our algorithm)	100%	95%	93%

**Table 5:** Comparison of various algorithms for coverage tasks.

Algorithm	Path Length	Coverage (%)	Redundancy (%)	Adaptability (%)	Complexity
Boustrophedon	237	87	18.4	60	Low
A* Coverage Algorithm	212	95	32.7	65	Low
DQN	189	82	28.2	65	Moderate
DDQN	178	84	27.4	75	Moderate
Dueling DQN	183	81	26.3	65	Moderate
PPO	173	93	11.4	85	very high
Re-DQN(Our algorithm)	159	95	6.2	90	Moderate

## Data Availability

The study did not report any data.
